# Operationalizing One Health in Saudi Arabia: a mixed-methods framework for national implementation

**DOI:** 10.3389/fpubh.2026.1816883

**Published:** 2026-06-19

**Authors:** Hessah Ibrahim Al Suwaidan, Asma Abdulaziz AlHossini, Sarah Ibrahim Alsuwaidan, Sulaiman Bah, Arwa Althumairi

**Affiliations:** 1 Health Information Management and Technology Department, College of Public Health, Imam Abdulrahman Bin Faisal University, Dammam, Saudi Arabia; 2 Department of Statistics and Information, Saudi Central Board for Accreditation of Healthcare Institutions, Riyadh, Saudi Arabia; 3 College of Business Administration, Princess Nourah Bint Abdulrahman University, Riyadh, Saudi Arabia

**Keywords:** framework development, implementation policy, One Health, public health, Saudi Arabia

## Abstract

**Background:**

The One Health (OH) approach is a method of integrating animal, environmental, and human health. Recently, there has been an international response to addressing zoonotic infections, antimicrobial resistance (AMR) and ecological hazards. The OH system in Saudi Arabia is in a positive position, though it still faces challenges in policy integration. The study aims to examine OH awareness, collaboration, implementation, and institutional roles, and to propose a national framework for OH implementation in Saudi Arabia.

**Methods:**

The study used an explanatory sequential mixed-methods design. A cross-sectional survey (*n* = 400) was administered to assess awareness of OH, collaboration, and implementation among health-related professionals and students. Chi-square tests, regression and Cohen’s kappa reflected the relationships and the degree of consensus on study variables. After that, semi-structured interviews were conducted to explore systemic aspects of barriers and facilitators. This support the establish of the proposed national OH operational structure.

**Results:**

The survey revealed relatively low OH awareness (81% had no prior knowledge), low collaboration (mean = 0.51; range = 0–4), and poor concurrence regarding the institutional roles of zoonotic diseases (*κ* = −0.13 to −0.10, all *p* < 0.001). Implementation was also correlated with OH awareness level (*R*^2^ = 0.167, *p* < 0.001). Age, higher levels of education, and profession influence awareness levels, according to regression analysis. Qualitative themes pointed to collaboration within the Saudi health system, with challenges reflected in government silos, gray routes in data, and institutional dispersion. All this, combined, led us to a multi-level OH framework, comprising national coordination, cross-sector functions and quantifiable program outputs.

**Conclusion:**

OH, in Saudi Arabia, is underdeveloped due to a lack of awareness, institutional misalignment, and integrated governance. We have a logical operational model, shaped by mixed-methods evidence and based on OH principles, that can inform policy, foster intersectoral collaboration, and streamline national implementation plans of action.

## 1 Introduction

Human, animal, and environmental health are interconnected components affected by complex global threats, such as zoonotic diseases, antimicrobial resistance (AMR), climate change, and food insecurity. The cross-disciplinary One Health (OH) approach is a collaborative framework across human, animal, and environmental sectors is increasingly receiving global attention for preventing and managing complex threats and reinforced the integrated and multisectoral approaches to public health governance (1–3). Despite the growing recognition of OH by international organizations such as the World Health Organization (WHO), Food and Agriculture Organization (FAO), World Organization for Animal Health (WOAH) and United Nations Environment Program (UNEP), OH implementation remains inconsistent across many countries due to fragmented governance structures, weak intersectoral coordination, and limited data exchange into public health systems (4–6).

OH is particularly relevant to Saudi Arabia due to its unique ecological, demographic, and public health context. Saudi Arabia occupies a strategic position within the Eastern Mediterranean Region (EMR) because of large-scale population mobility, mass gathering events such as Hajj and Umrah, extensive livestock movement, and diverse environmental settings, all of which increase the risk of zoonotic disease transmission and emerging infectious threats (7–9). In addition, Vision 2030’s objectives related to environmental sustainability, food security, digital transformation, and health system strengthening are closely aligned with OH principles (10, 11). However, despite this strategic relevance, OH-related activities in Saudi Arabia continue to operate largely in institutional silos across ministries, universities, and independent research sectors, limiting the development of coordinated national OH governance mechanisms (12).

Most Saudi-based research has focused primarily on specific illnesses, AMR surveillance, and environmental hazards, rather than examining OH as an integrated governance and operational framework (13–15). Similarly, while several international OH frameworks and conceptual guidance models have been proposed, relatively few studies have developed evidence-based, context-specific, and operational national OH implementation models informed by stakeholder perspectives and mixed-methods evidence (16–18).

To address these gaps, we employed an explanatory sequential mixed-methods design to examine OH awareness, collaboration, implementation, and institutional roles in Saudi Arabia, and to develop a proposed national framework for OH implementation.

## 2 Methods

### 2.1 Study design

The study employed an explanatory sequential mixed-methods research design guided by the principles of systems thinking. The quantitative phase was conducted first to develop a baseline understanding of OH awareness, collaboration, and implementation patterns. The qualitative phase subsequently explored the underlying institutional, structural, and governance factors shaping these patterns the integration of both phases supported the development of an evidence-based national OH implementation framework (19, 20).

### 2.2 Framework context

**Figure 1:**
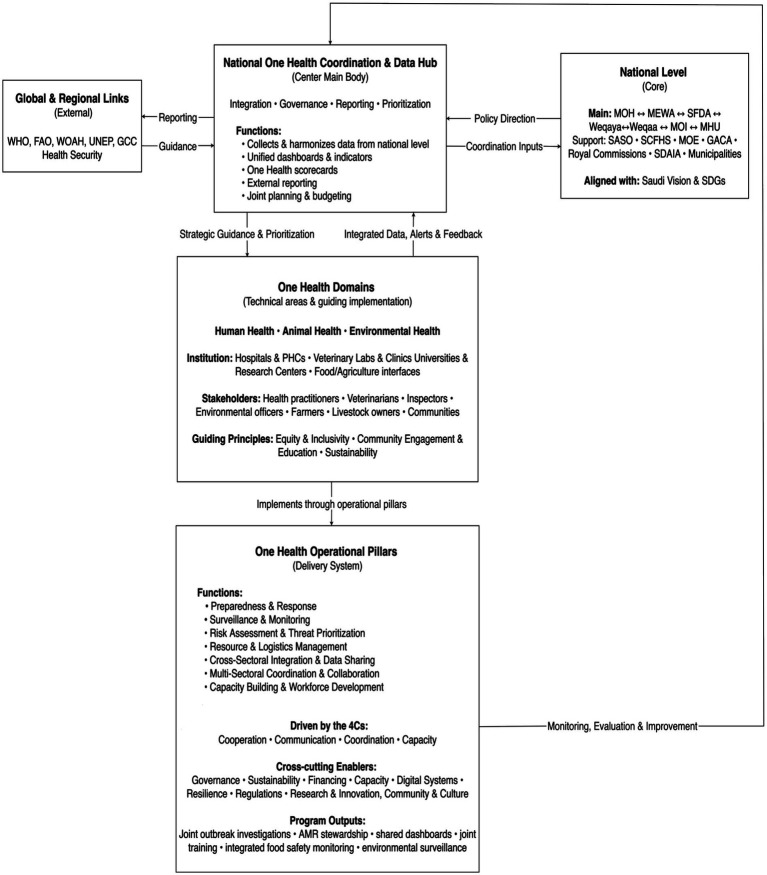
The proposed national One Health operational framework in Saudi Arabia.

The proposed Saudi OH framework is a functional integration framework, not merely a conceptual diagram. It was constructed inductively by interweaving national policy focus, the international OH policy guidance (WHO, FAO, WOAH, UNEP), and data from both quantitative findings and qualitative thematic results. To enhance contextual validity, the framework was reviewed by experts for contextual relevance and applicability from the human, animal, and environmental sectors through a structured consultation process that reviewed clarity, feasibility, and applicability using structured feedback forms (21). The framework incorporates systems-thinking concepts, coordination pathways, institutional roles, and enabling mechanisms relevant to OH implementation in Saudi Arabia (22, 23). The experts reviews proved useful for the model’s structure, terminology, and directional connections within the framework, leading to refinement of the final framework presented in Figure 1.

### 2.3 Quantitative phase

#### 2.3.1 Instrument development

The quantitative instrument was adapted from previously validated OH instruments assessing awareness, collaboration, and governance capacity (24). The questionnaire domains included demographics information, OH awareness, institutional collaboration, role clarity, and perceived implementation barriers.

Content and face validity were assessed by five subject experts from public health, veterinary and environmental disciplines. A pilot study (n = 30) was conducted among participants with similar characteristics to the target population to evaluate clarity, cultural relevance, and item interpretation and minor wording refinements were applied. Internal consistency reliability was acceptable across domains (Cronbach’s *α* = 0.71–0.83).

#### 2.3.2 Sampling and participants

A non-probability convenience sampling strategy was used to capture variability across OH-related sectors. The target group comprised students, trainees, and professionals in the human, veterinary, and environmental health sectors at university settings and health research centers in Saudi Arabia.

The final sample size was n = 400, calculated using the single-proportion formula (95% confidence level, 5% margin of error, *p* = 0.5) (25). Assuming 50% awareness, 95% confidence, and 5% precision, the minimum required sample size was estimated at 385 participants.

$$n=\frac{\left(Z^{2})\times p\times (1-p\right)}{d^{2}}=\frac{{\left(1.96\right)}^{2}\times 0.50\times (1-0.50)}{{\left(0.05\right)}^{2}}\approx 385.$$

#### 2.3.3 Data collection and management

The data were collected via Google Forms and via face-to-face QR code scanning between January and April 2025. The involvement was voluntary and anonymous. The data was exported into Microsoft Excel, cleaned, coded, and prepared for analysis. All questionnaires were completed electronically; incomplete responses (<5% of dataset) were excluded during cleaning and no significant non-response bias was observed.

#### 2.3.4 Quantitative data analysis

The analysis involved descriptive and inferential statistical measures using SPSS v23. Descriptive statistics summarized OH awareness and collaboration levels. The chi-square test examined associations between categorical variables. Cohen’s kappa tested the agreement of institutional roles related to zoonotic diseases. Multivariate linear regression was used to estimate predictors of OH awareness after assessment of model assumptions and collinearity. Linear regression was considered appropriate because the OH awareness score demonstrated an approximately continuous distribution. Model assumptions were assessed using normality of residuals (Q-Q plots and Shapiro–Wilk test), multicollinearity (VIF < 5), homoscedasticity (residual plots), and independence of errors (Durbin-Watson statistic). The maintained statistical significance of the *p*-value of less than 0.05 (two-tailed).

### 2.4 Qualitative phase

#### 2.4.1 Sampling and recruitment

Purposive sampling was used to ensure representation across OH sectors and institutional roles. A total of seven high-level institutional experts from national health institutions and universities participated in the qualitative phase. This sampling strategy enhanced the diversity of institutional perspectives relevant to OH governance (26).

#### 2.4.2 Data collection

Semi-structured interviews were conducted with the senior experts through Microsoft Teams for around 45–60 min. The interviews were audio-taped with permission and transcribed professionally. To enable data contextualisation, we collected brief demographic data (sector, role, institutional type, years of experience) and maintained anonymity. The interview guide was based on the quantitative research results, which provided inquiries into the lack of collaboration, negative tendencies toward agreement, disparities between awareness and practice, unclear institutional requirements, and opportunities to improve OH systems.

#### 2.4.3 Qualitative analysis

Data saturation was assessed through iterative coding and comparison across transcripts. There were no new themes emerged after the seventh interview, indicating thematic redundancy within the defined experts sample. The data were analyzed using reflexive thematic analysis (Braun & Clarke approach) supported by NVivo software (27). The study carried out an inductive analysis through data familiarization, initial coding, theme development and review and refinement of themes. The themes were examined in terms of feedback loops, power structures, coordination bottlenecks, and leverage points. Analytic memos enhanced transparency, while credibility was strengthened through reflexivity, peer discussion, and repeated comparison across transcripts.

### 2.5 Integration

This section follows an explanatory sequential reasoning approach, in which the initial quantitative results informed the design and the questions included in the qualitative section. This enabled deeper exploration of emerging quantitative patterns (see Table 1). We first compare quantitative and qualitative data independently and then align the results through side-by-side analysis, joint displays, and an ongoing process of analytic dialog. We systematically searched for convergence, complementarity, and expansion to arrive at higher inferences. Integration was done at three levels:

1. Design level - quantitative findings informed qualitative sampling and interview development.
2. Methods level- alignment of quantitative variables, with qualitative codes and themes at phases.
3. Interpretative level - incorporating results to inform the national development of OH framework.

**Table 1:** Overview of the explanatory sequential mixed-methods design.

Component	Phase 1: Quantitative	Phase 2: Qualitative
Priority and sequence	Quantitative first, then qualitative	Builds on quantitative findings
Purpose	Describe OH awareness, roles, and collaboration	Explain patterns and refine the framework
Study design	Cross-sectional survey	Semi-structured interviews
Sampling strategy	Convenience sampling	Purposeful sampling
Sample size	*n* = 400	*n* = 7
Setting	Universities & health research center	National institutions and universities
Data collection method	Self-administered structured questionnaire adapted from the validated One Health instrument	Semi-structured interview guide informed by Phase 1 results; conducted via Microsoft Teams
Data analysis	Descriptive and inferential statistics in SPSS v23	Reflexive thematic analysis, iterative coding in NVivo; theme mapping
Ethical and rigorous procedures	IRB approval; informed consent; anonymity; validity (Cronbach’s α > 0.7)	IRB approval; informed consent; confidentiality; audit trail; peer checking; reflexivity; transparency

This has helped ensure that the construction and framework development and interpretations were grounded in empirical findings rather than preliminary assumptions, in line with most acceptable practices in explanatory sequential mixed-methods research (19, 20).

### 2.6 Ethical considerations

The IRB provided ethical approval from Imam Abdulrahman Bin Faisal University (IRB-PGS-2024-03-464). Informed consent was obtained from all the participants; we maintained confidentiality and privacy procedures.

## 3 Results

### 3.1 Quantitative results

The sample for the quantitative stage consisted of 400 participants (94.3% were female, and 95.3% were Saudi citizens). The largest age group was 18–24 (88.5%). Most respondents (89.8 and 88.8%, respectively) were members of academic institutions and undergraduates which should be considered when interpreting the findings beyond university-based populations. Their primary fields of learning included public health, medicine, and the sciences. Within the study sample, awareness of OH was relatively low with 81 per cent reported no prior knowledge of the phenomenon, 13.4 per cent reported limited knowledge, and less than 6 per cent reported moderate or high knowledge (Table 2).

**Table 2:** Baseline demographic characteristics of study participants (*N* = 400).

Characteristics		*n* (%)
Sex		
	Female	377 (94.3)
	Male	23 (5.8)
Age		
	18–24 years	354 (88.5)
	25–34 years	20 (5)
	35–44 years	19 (4.8)
	45–54 years	5 (1.3)
	55 years and above	3 (0.5)
Nationality		
	Saudi	381 (95.3)
	Non-Saudi	19 (4.8)
Place of residence		
	Northern Region	16 (4)
	Central Region	320 (80)
	Southern region	9 (2.3)
	Eastern Region	42 (10.5)
	Western Region	13 (3.3)
Academic degree		
	Intermediate or secondary	101 (25.3)
	Bachelor	270 (67.5)
	Masters	15 (3.8)
	PhD	14 (3.5)
Current professional		
	Undergraduate students	355 (88.8)
	Graduate students	10 (2.5)
	Medical professionals	13 (3.3)
	Specialized professionals	15 (3.8)
	Academic professionals	7 (1.8)
Specialization		
	Public health	80 (20)
	Human medicine and dentistry	168 (42)
	Environmental sciences	9 (2.3)
	Rehabilitation and allied health sciences	13 (3.3)
	Sciences	43 (10.8)
	Pharmacy and medical laboratories	16 (4)
	Veterinary science	8 (2)
	Nursing	63 (15.8)
Type of institution		
	Government/ministry institution	24 (6)
	Academic institution	359 (89.8)
	Private sector	11 (2.8)
	Research institution	6 (1.5)
Knowledge level One Health		
	No knowledge	324 (81)
	Limited knowledge	54 (13.4)
	Moderate knowledge	17 (4.3)
	Advanced knowledge	5 (1.3)

The level of awareness varied among various sociodemographic attributes. There was a relationship between the OH level of awareness and gender, age, academic degrees, current professional and type of institution (*p* < 0.05). Higher OH awareness levels were observed among participants with higher professional exposure and advanced academic qualifications (Table 3).

**Table 3:** Relationship between participant characteristics and One Health knowledge using Chi-square tests.

Characteristics	Familiarity with One Health knowledge		*p*-value
	Yes *n* (%)	No *n* (%)	
Sex			0.005†
Female	66 (17.5)	311 (82.5)	
Male	10 (43.5)	13 (56.5)	
Age			0.000†
18–24 years	53 (15)	301 (85)	
25–34 years	12 (60)	8 (40)	
35–44 years	8 (42.1)	11 (57.9)	
45–54 years	2 (40)	3 (60)	
55 years and above	1 (50)	1 (50)	
Nationality			0.049†
Saudi	69 (18.1)	312 (81.9)	
Non-Saudi	7 (36.8)	12 (63.2)	
Place of residence			0.137
Northern Region	2 (12.5)	14 (87.5)	
Central Region	56 (17.5)	264 (82.5)	
Southern region	1 (11.1)	8 (88.9)	
Eastern Region	12 (28.6)	30 (71.4)	
Western Region	5 (38.5)	8 (61.5)	
Academic degree			0.000†
Intermediate or secondary	11 (10.9)	90 (89.1)	
Bachelor	49 (18.1)	221 (81.9)	
Masters	9 (60)	6 (40)	
PhD	7 (50)	7 (50)	
Current professional			0.000†
Undergraduate students	54 (15.2)	301 (84.8)	
Graduate students	4 (40)	6 (60)	
Medical professionals	7 (53.8)	6 (46.2)	
Specialized professionals	7 (46.7)	8 (53.3)	
Academic professionals	4 (57.1)	3 (42.9)	
Specialization			0.108
Public health	22 (27.5)	58 (72.5)	
Human medicine and dentistry	26 (15.5)	142 (84.5)	
Environmental sciences	2 (22.2)	7 (77.8)	
Rehabilitation and allied health sciences	0 (0)	13 (100)	
Sciences	10 (23.3)	33 (76.7)	
Pharmacy and medical laboratories	4 (25)	12 (75)	
Veterinary science	3 (37.5)	5 (62.5)	
Nursing	9 (14.3)	54 (85.7)	
Type of institution			0.000†
Government/ministry institution	12 (50)	12 (50)	
Academic institution	57 (15.9)	302 (84.1)	
Private sector	5 (45.5)	6 (54.5)	
Research institution	2 (33.3)	4 (66.7)	

†Significant *p*-value is ≤0.05.

Respondents reported mean scores indicating a little developed collaboration process (*M* = 0.51) and comparatively low numbers of strategies for collaboration toward OH (*M* = 0.88). The rating of perceived barriers was relatively elevated (*M* = 3.56). The respondents reported an average of 3.36 gaps in OH existing programs, and the institutional initiatives toward OH had a mean of 2.89 (Table 4).

**Table 4:** Descriptive statistics of factors and challenges related to One Health among study participants.

Variable	Mean	SD	Minimum	Maximum
Professionals involved in One Health initiatives	3.37	2.655	0	8
Factors of diseases common to humans and animals from environmental toxicants	5.02	2.874	0	9
Factors exacerbating antimicrobial resistance	3.45	1.526	0	6
Factors limiting collaboration in One Health	3.56	2.376	0	9
One Health collaboration level	0.51	1.062	0	4
Strategies for One Health collaboration	0.88	2.000	0	8
Institutions responsible for One Health activities	3.93	2.533	0	9
Gaps identified in One Health initiatives	3.36	2.177	0	8
Initiatives to promote One Health awareness	2.89	1.558	0	5

**Figure 2:**
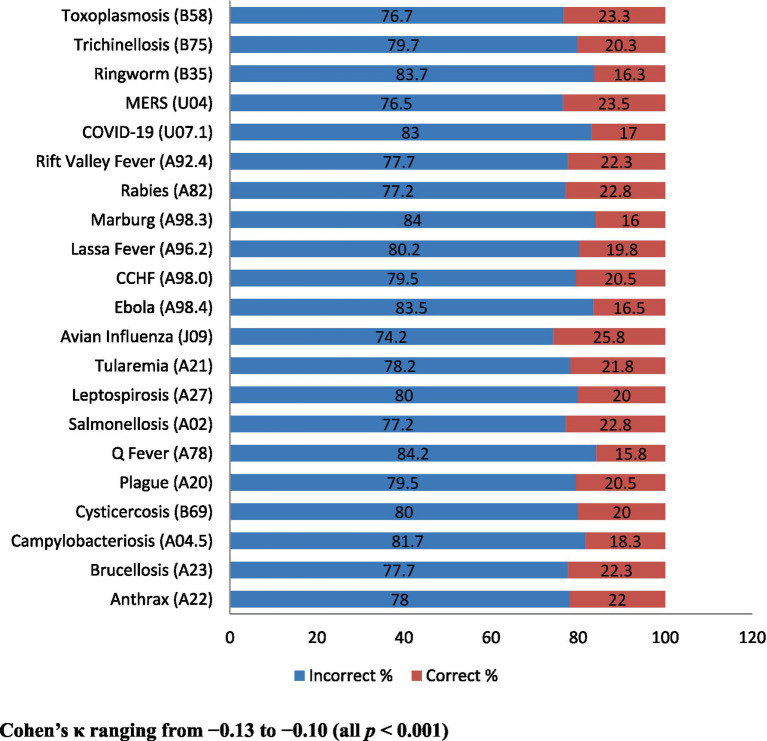
Agreement of understanding institutional responsibilities for zoonotic diseases using Cohen’s Kappa coefficient. Cohen’s κ ranging from −0.13 to −0.10 (all *p* < 0.001).

Figure 2 shows the agreement between institutional responsibilities and zoonotic diseases. The observation reflected a misalignment of responsibilities among the ministries; respondents often grouped diseases under a single agency rather than assigning them to multiple agencies. The correct attribution ranged from 15.8 to 25.8%. The value of Cohen’s kappa showed negative consistency across most disease groups (*κ* = −0.13 to −0.10, all *p* < 0.001), indicating agreement below chance level and reflecting substantial inconsistency in participants’ understanding of institutional responsibilities. These findings suggest unclear institutional boundaries and fragmented perceptions regarding OH governance responsibilities.

The respondents acknowledge the potential benefits of OH. The highest ratings were increased disease control and response to an outbreak (*M* = 3.95), enhanced health and well-being (*M* = 3.95), and enhanced information quality (*M* = 3.83). The lowest scores were found for antimicrobial resistance surveillance (*M* = 2.62), environment toxicant-associated illnesses (*M* = 2.79), and the presence of national OH programs (*M* = 2.57). The implementation findings indicate a range of means from 2.75 to 2.97 for OH professionals’ involvement, university OH curricula, and engagement, but OH institution regulation showed a mean of 3.18 for operational aspects (Table 5).

**Table 5:** Evaluating the level of perception between the awareness and implementation scale scores of One Health perception in Saudi Arabia.

Variable		Mean	SD	Minimum	Maximum
Awareness of One Health approach					
1.	Early detection of threats	3.63	1.301	1	5
2.	Controlling diseases with better response	3.95	1.178	1	5
3.	Economic benefits	3.41	1.287	1	5
4.	Improving health and well-being	3.95	1.145	1	5
5.	High-quality information	3.83	1.210	1	5
6.	Environmental benefits	3.71	1.253	1	5
7.	Personal or social benefits	3.73	1.244	1	5
8.	Health policy design	3.80	1.180	1	5
9.	Promoting research and development	3.78	1.197	1	5
10.	Strengthening international cooperation	3.66	1.249	1	5
11.	Awareness of common diseases between humans and animals caused by environmental toxicants	2.79	1.103	1	5
12.	Awareness of antimicrobial resistance issues through surveillance and research programs	2.62	1.062	1	5
13.	Awareness of the One Health approach in Saudi Arabia	2.57	1.155	1	5
Implementation of the One Health approach					
1.	Participation in events involving veterinarians, human physicians, and environmental scientists	2.80	1.094	1	5
2.	Inclusion of subjects related to veterinary, medical, and environmental sciences in university curricula	2.75	1.123	1	5
3.	Existence of institutions or ministries regulating or managing veterinary control and food chains	2.97	1.110	1	5
4.	Existence of institutions or ministries handling environmental emergencies and community resilience	3.18	1.147	1	5
5.	Implementation of the One Health across the community, veterinary, and environmental health sectors	2.94	1.056	1	5

**Figure 3:**
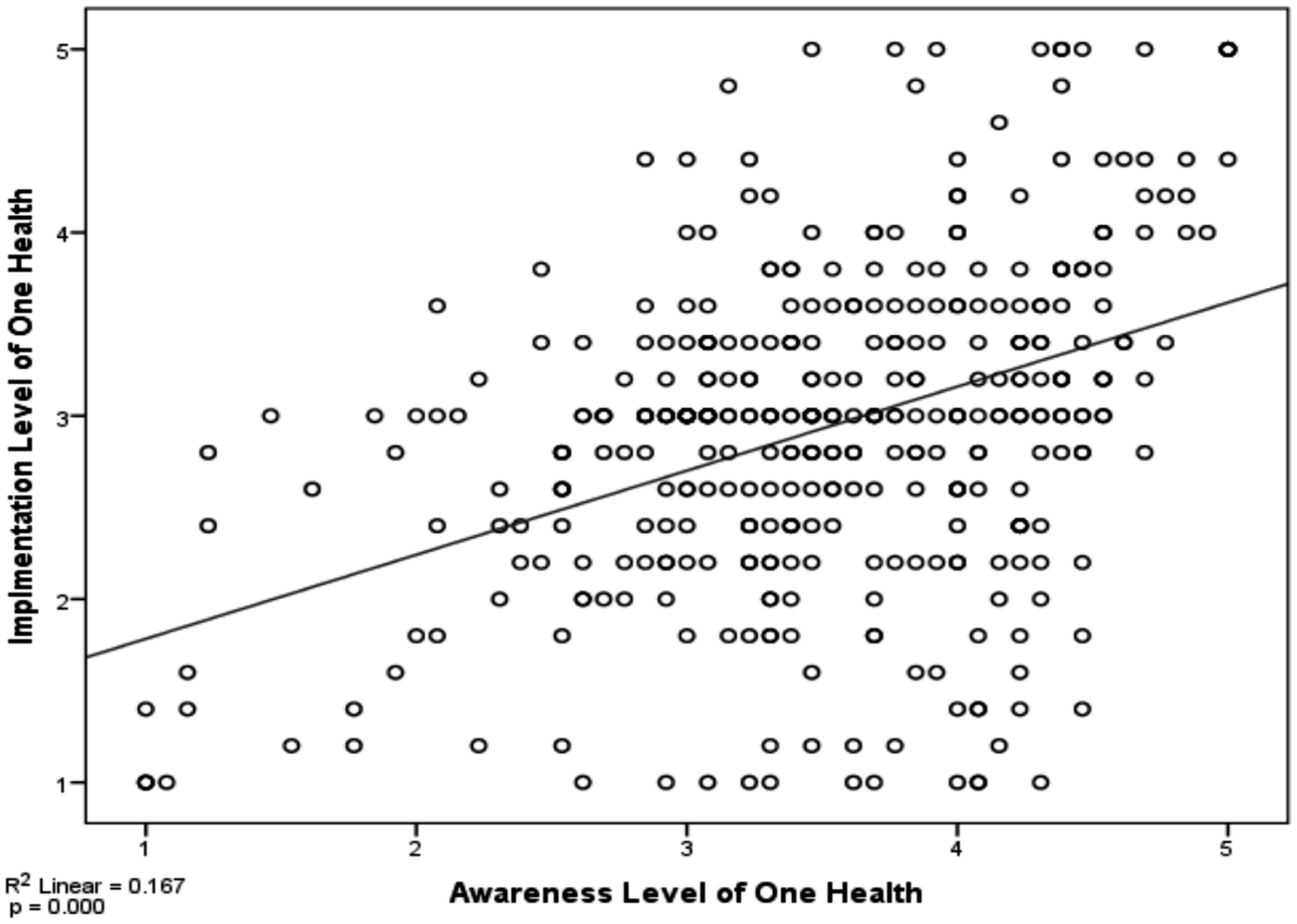
Scatterplot showing the relationship between One Health awareness and implementation scores.

The coefficients of a scatter-plot analysis showed that the level of implementation and awareness of OH principles had a moderate, positive relationship (*R*^2^ = 0.167, *p* = 0.000). Specifically, higher awareness scores were moderately associated with higher perceived implementation scores (Figure 3).

The multivariate regression revealed that OH awareness scores were significantly higher among respondents aged 25–34 years (*p* = 0.005), men (*p* = 0.024), master’s degree holders (*p* = 0.000), and academic professionals (*p* = 0.029). Enhanced professional exposure and higher levels of training are correlated with better conceptual understanding of OH. Conversely, academic institution affiliation was negatively correlated with awareness levels (*p* = 0.019), suggesting lower awareness scores among participants affiliated with academic institutions compared with government/ministry institutions. It was also observed that nursing specialization was negatively associated with OH awareness scores (*B* = −0.300, *p* = 0.006). Although several predictors reached statistical significance, the model demonstrated limited explanatory power, indicating that OH awareness is likely influenced by additional unmeasured contextual and institutional factors (Table 6).

**Table 6:** Multivariable regression analysis of One Health awareness score and demographic characteristics.

Characteristic	Groups	*B*	*p*-value	95% CI
Sex	(Ref: Female) ‡			
	Male	0.385	0.024†	[0.051 to 0.718]
Age	(Ref: 18–24 years) ‡			
	25–34 years	0.505	0.005†	[0.149 to 0.860]
	35–44 years	0.323	0.083	[−0.043 to 0.689]
	45–54 years	0.388	0.278	[−0.314 to 1.091]
	55 years and above	0.586	0.298	[−0.521 to 1.693]
Nationality	(Ref: Saudi) ‡			
	Non-Saudi	0.009	0.963	[−0.359 to 0.376]
Place of Residence	(Ref: Northern Region) ‡			
	Central Region	−0.085	0.393	[−0.280 to 0.110]
	Southern Region	−0.243	0.365	[−0.769 to 0.284]
	Eastern Region	0.208	0.109	[−0.047 to 0.462]
	Western Region	0.163	0.469	[−0.278 to 0.603]
Academic degree	(Ref: Intermediate or Secondary) ‡			
	Bachelor	−0.118	0.164	[−0.206 to 0.154]
	Masters	0.771	0.000†	[0.367 to 1.176]
	PhD	0.086	0.690	[−0.339 to 0.512]
Current professional role ‡	(Ref: Undergraduate Students)			
	Graduate Students	0.480	0.059	[−0.019 to 0.978]
	Medical Professionals	0.150	0.503	[−0.290 to 0.591]
	Specialized Professionals	0.313	0.135	[−0.097 to 0.723]
	Academic Professionals	0.661	0.029†	[0.068 to 1.253]
Specialization	(Ref: Public Health) ‡			
	Human Medicine & Dentistry	0.120	0.136	[−0.038 to 0.278]
	Environmental Sciences	0.212	0.429	[−0.315 to 0.739]
	Rehabilitation & Allied Health Sciences	0.095	0.671	[−0.346 to 0.536]
	Sciences	0.050	0.696	[−0.202 to 0.303]
	Pharmacy & Medical Laboratories	0.047	0.818	[−0.352 to 0.446]
	Veterinary Science	0.320	0.259	[−0.237 to 0.878]
	Nursing	−0.300	0.006†	[−0.513 to −0.088]
Type of institution	(Ref: Government/Ministry Institution) ‡			
	Academic Institution	−0.307	0.019†	[−0.563 to −0.051]
	Private Sector	0.398	0.101	[−0.078 to 0.875]
	Research Institution	0.163	0.619	[−0.480 to 0.806]

† Significant *p*-value is ≤0.05. Model fit (Full model): Adjusted *R*^2^ = 0.024. ‡ Reference categories for dummy-coded variables were selected based on the lowest value or first group in the respective category list: Female (Sex), 18–24 years (Age), Saudi (Nationality), Northern Region (Place of Residence), Intermediate or Secondary (Academic Degree), Undergraduate Students (Current Professional Role), Public Health (Specialization), and Government/Ministry Institution (Type of Institution).

A parsimonious model was developed using backward-stepwise regression (see Table 7). The awareness scores were observed significantly among individuals aged 25–34 (*B* = 0.489, *p* = 0.007). OH awareness was significantly elevated among graduate students (*B* = 0.528, *p* = 0.036) and academic professionals (*B* = 0.626, *p* = 0.036), while a significant negative association was observed with nursing specialization (*B* = −0.288, *p* = 0.009). Although these predictors were significant in the final model, the model fit was poor (Adjusted *R*^2^ = 0.07) and accounted for only a limited proportion of the variation in awareness scores. All other demographic variables, such as sex, nationality, region of residence, academic degree, and institutional type, were not statistically significant and were removed from the final model during the backward elimination process.

**Table 7:** Regression analysis predicting One Health awareness scores by the parsimony model.

Predictor variable		B (Unstd.)	Std. Error	β (Std.)	t	*p*	95% CI for B
Age group	Age 25–34 years (Ref: 18–24)	0.489	0.180	0.134	2.721	0.007†	[0.136 to 0.843]
Current professional role	Graduate students (Ref: Undergraduate Students)	0.528	0.251	0.104	2.107	0.036†	[0.035 to 1.021]
	Academic Professionals (Ref: Undergraduate Students)	0.626	0.297	0.103	2.108	0.036†	[0.042 to 1.210]
Specialization	Nursing (Ref: Public Health)	−0.288	0.109	−0.132	−2.629	0.009†	[−0.503 to −0.073]

† Significant *p*-value is ≤0.05. Model fit (Final backward model): Adjusted *R*^2^ = 0.07; F (df = 4, 395) ≈ 7.0; *p* < 0.001.

### 3.2 Qualitative results

#### 3.2.1 Participant characteristics

The data used in the qualitative phase were provided by seven senior experts in human, animal, and environmental health. There were five males and two females. Four of them worked at national health agencies, whereas the other three were academics from government universities. The main areas covered by OH activities are frontline activities, instruction, curriculum development, and research. Most participants had distinguished professional experience; Most participants had substantial professional experience, with six of the seven participants reporting more than 10 years of experience, which providing experienced system-level perspectives on OH governance and implementation. Both Saudi and non-Saudi experts contributed a variety of professional backgrounds and experiences to the study.

Reflexive notes were made immediately after the interviews, showing the richness of the context. Other informants initially seemed conservative but provided increasingly detailed reflections as the interview unfolded, producing more detailed information about institutional dynamics. While others required clarification prompts to keep the study goals in focus, and in a few interviews, differences in terminology familiarity or conceptual interpretation occasionally resulted in repetitive or less detailed responses. These differences were considered in the analysis and did not change the underlying themes; instead, they helped clarify how sectoral stance, familiarity with OH, and communicative ease influence the depth of the narrative (Table 8).

**Table 8:** Characteristics of One Health qualitative interview participants (*N* = 7).

Characteristics	*n* (%)
Sex	
Female	2 (28.6)
Male	5 (71.4)
Primary sector	
Human health	3 (42.9)
Animal / veterinary health	3 (42.9)
Environmental health	1 (14.3)
Institution type	
Government/national authority	4 (57.1)
University/research institution	3 (42.9)
Nationality	
Saudi	4 (57.1)
Non-Saudi	3 (42.9)
Years of experience	
6–10 years	1 (14.3)
11–20 years	3 (42.9)
> 20 years	3 (42.9)

#### 3.2.2 Thematic findings

The quantitative findings informed the subsequent qualitative phase, particularly regarding the observed gaps in institutional coordination, low awareness levels, and inconsistencies in perceived OH responsibilities across sectors. The results of the coding analysis provided four themes as shown in Table 9: (1) intersectoral collaboration dynamics; (2) governance and coordination structures; (3) information systems and technical integration; (4) education, community, and sectoral engagement.

**Table 9:** Illustrative coding framework showing progression from raw quotes to themes.

Raw quote	Code	Category	Final analytic theme
“From this level of collaboration, you can clearly see that cooperation between us is very strong. Yes, there are challenges and obstacles, but in terms of communication and intergovernmental cooperation, I see it as truly excellent.” P3	Strong collaboration reported, alongside operational challenges	Mixed collaboration performance	Intersectoral collaboration dynamics
“Coordination between other entities depends on the nature of the work. Some diseases have their own dedicated committee…” P3	Collaboration concentrated in specific program areas	Episodic coordination	
“Currently, if I want data, I have to communicate with someone, who then connects me to another person, etc.—which is a problem.” P4	Coordination facilitated through informal networks	Informal coordination mechanisms	
“Where the leadership believes in One Health, things move faster.” P2	Leadership engagement linked to implementation momentum	Institutional drivers of progress	
“AMR is also overseen by a multisectoral committee that includes environment, animal health, and public health.” P3	Cross-sector AMR coordination example	Policy coordination	
“Each sector has the authority and the right to set its own priorities and develop its own programs… We aligned both plans… and we meet at the end of each quarter or each year… to review what has been accomplished.” P3	Independent sectoral planning with periodic alignment	Distributed coordination arrangements	Governance and coordination structures
“The most prominent challenge in One Health is the differences in regulations… This is why I believe that regulatory differences are the main challenge.” P3	Lack of clarity around operational intersection	Governance pathways are not fully defined	
“.translated into clear vocal point and some measurable… deliverables that could actually measure how well we work with this entity?” P2	Interest in structured accountability tools	Governance tools for alignment and accountability	
“Information sharing—definitely… There must be a platform… A unified platform… So, the biggest challenge is information exchange.” P4	Information sharing is identified as an operational constraint	Data-sharing and interoperability issues	Information systems and technical integration
“There must be a platform that allows us to access data—with controlled access—and vice versa. A unified platform.” P4	Perceived need for integrated digital platforms	Data fragmentation	
“We need a national platform that connects data… and in the future, AI can help us see risks before they spread.” P7	Forward-looking interest in digital integration and analytics	Future-oriented technical systems	
“Currently, if I want data, I have to communicate with someone… which is a problem.” P4	Data access routed through interpersonal channels	Interoperability gaps	
“Here, all sectors are involved. I cannot take full control on my own!. Any overlap between my priorities becomes a shared responsibility.” P3	Joint accountability with unclear ownership governance	Cross-sector role ambiguity	
“Universities incorporating some of the training within the curriculum will be very helpful… I think we need to engage universities, specifically, much earlier.” P2	Limited translation from educational concepts to institutional practice	Training and application gaps	Education, community, and sectoral engagement
“The universities really are relying on the traditional methods of just having the priorities laid down from years ago and not being agile to new areas.” P2	Established academic planning processes with slower adaptation cycles	Structural and institutional constraints	
“Farmer owners and rural communities must be engaged… they are the first to see animal diseases before anyone else.” P1	Value placed on community-level engagement	Community participation opportunities	
“When the practices of humans, animals, and plants are aligned and functioning well, the ecosystems will naturally become healthier.” P3	Opportunities to broaden the environmental health scope	Expanding environmental perspectives beyond a biomedical focus	
“All of them are active. Maybe in past periods, the environment sector… its engagement was limited in some tracks — not across all One Health domains.” P3	Engagement is heterogeneous across domains and has developed over time	Fragmented but evolving participation	

### 3.3 Theme

#### 3.3.1 Intersectoral collaboration dynamics

Participants described collaboration as present but variable across sectors and programs. Collaboration was also perceived as dynamic and enhancing, but it occurred more certainly on specific tracks rather than along integrated pathways.

“*From this level of collaboration, you can clearly see that cooperation between us is very strong. Yes, there are challenges and obstacles, but in terms of communication and intergovernmental cooperation, I see it as truly excellent*”. P3.

“*Coordination between other entities depends on the nature of the work. Some diseases have their own dedicated committee…*” P3.

Professional connections were effective in promoting data-sharing coordination, whereas formal systems render processes reliant on networks.

“*Currently, if I want data, I have to communicate with someone, who then connects me to another person, etc.—which is a problem*.” P4.

Leadership involvement was also reported as being a significant facilitating element for faster OH involvement:

“*Where the leadership believes in One Health, things move faster*.” P2.

The combination of these views indicates a system of cooperation, although it is yet to be standardized.

### 3.4 Theme

#### 3.4.1 Governance and coordination structures

The participants indicated governance systems in which the sectors maintain power over their respective mandates, and that periodic alignment procedures are in place. This model was perceived as a realistic way and supportive of the institutional roles.

“*Each sector has the authority and the right to set its own priorities and develop its own programs… We aligned both plans… and we meet at the end of each quarter or each year… to review what has been accomplished*.” P3.

Meanwhile, some respondents also noted that operational intersections within cross-sectoral arrangements were not always well-defined, especially in day-to-day operations.

“*The most prominent challenge in One Health is the differences in regulations… This is why I believe that regulatory differences are the main challenge*.” P3.

It was evidently interested in instruments that could formalize coordination, such as platforms, common indicators, and formal review processes, although they were not intended to obliterate sectoral independence.

“*...translated into clear vocal point and some measurable… deliverables that could actually measure how well we work with this entity?*” P2.

Overall, participants described governance arrangements that attempt to balance sectoral independence with cross-sector coordination, although operational coherence remained inconsistent.

### 3.5 Theme

#### 3.5.1 Information systems and technical integration

The most frequently reported operational limitations are information sharing. The participants referred to the information systems as incoherent, and data often goes to individual organizations rather than integrated systems.

“*Information sharing—definitely… There must be a platform… A unified platform… So, the biggest challenge is information exchange*.” P4.

“*Currently, if I want data, I have to communicate with someone… which is a problem*.” P4.

A unified digital system, real-time dashboards, and analytical tools, such as artificial intelligence (AI), are recommended to support early detection and response, as well as data integration.

“*We need a national platform that connects data… and in the future, AI can help us see risks*.”

Although at the same time, pointing to uncertainty regarding leadership in the spheres.

“*AMR is also overseen by a multisectoral committee that includes environment, animal health, and public health*.” P3.

While simultaneously emphasizing leadership roles across sectors.

“*Here, all sectors are involved. I cannot take full control on my own!... Any overlap between my priorities becomes a shared responsibility*.” P3.

These findings suggest that integration mechanisms remain under development rather than fully institutionalized.

### 3.6 Theme

#### 3.6.1 Education, community, and sectoral engagement

The participants perceived the universities, communities and sectoral partners as underrepresented stakeholders in the OH system. Academic curricula were conceptually helpful in translating OH into practice.

“*Universities incorporating some of the training within the curriculum will be very helpful… I think we need to engage universities, specifically, much earlier*.” P2.

Participants perceived universities as operating within relatively fixed institutional structures that may adapt more slowly to emerging OH priorities.

“*The universities really are relying on the traditional methods of just having the priorities laid down from years ago and not being agile to new areas*.” P2.

The participants also made it clear that rural communities and farmers tend to notice the first signs of infection before institutions do, and that they should be involved.

“*Farmer owners and rural communities must be engaged… they are the first to see animal diseases before anyone else*.” P1.

The environmental sectors implied the possibility of expanding system participation and shifting the predominantly biomedical focus.

“*When the practices of humans, animals, and plants are aligned and functioning well, the ecosystems will naturally become healthier*.” P3.

The final insights identify actors who may play an important role in enhancing the OH system’s responsiveness.

### 3.7 Reflexivity

The qualitative part was carefully conducted, as the researchers in the field of public health recognized that their background and prior knowledge of OH might affect how we perceived the participants’ descriptions. To minimize this bias, analytic decisions were recorded in reflexive memos, interpretations were reviewed alongside raw transcripts, and efforts to identify common patterns did not override divergent perspectives. We also acknowledged that senior officials hold power and tried to privilege participants’ language in the process of creating themes and descriptions. These measures increased transparency and ensured that the results were based on participants’ experiences rather than on hypothesized models of how OH was supposed to work.

### 3.8 Integration of quantitative and qualitative findings

#### 3.8.1 Convergence: quantitative patterns confirmed by qualitative insight

The results of our survey showed a lack of formal awareness of OH: 81 per cent of respondents stated they had never heard the term, and less than 6 per cent indicated they were moderately or highly familiar with it. Simultaneously, the respondents were highly confident in the possibilities of OH, specifically in enhancing disease control (*M* = 3.95), population health and wellbeing (*M* = 3.95), and the quality of information (*M* = 3.83). The indicators of implementation were close to the central value, and collaboration ratings were low (*M* = 0.51), suggesting conceptual advocacy and the creation of working structures.

Interview narratives supported this picture. Respondents frequently explained OH as in its early stages of practice, with a tendency toward collaboration within program domains as opposed to happening on an everyday basis across systems:

“*Coordination between other entities depends on the nature of the work. Some diseases have their own dedicated committee…*” P3.

The domains of knowledge in the survey that were poorest included antimicrobial resistance (*M* = 2.62) and environmental toxicants (*M* = 2.79). The interviewees said that AMR was scattered in sectors, and it made clarifying leadership rather challenging at times:

“*AMR is also overseen by a multisectoral committee that includes environment, animal health, and public health*.” P3.

These findings converge on a common trend that OH is generally considered viable and suitable, and implementation processes continue to evolve, even amid some challenges. With integration, stakeholders are enthusiastic about the OH concept and recognize the correct course of action, but coordination issues and technical limitations hinder its full implementation.

### 3.9 Complementarity and explanation: qualitative insights clarify why patterns occur

According to the survey results, there was uncertainty about the roles of institutions in controlling zoonotic diseases, with low correct attribution and negative agreement (*κ* < 0, *p* < 0.001). According to interviewees, this confusion had more to do with the change in coordination structure than personal confusion:

“*Each sector has the authority and the right to set its own priorities and develop its own programs… We aligned both plans… and we meet at the end of each quarter or each year… to review what has been accomplished*.” P3.

The perceived barriers to collaboration (*M* = 3.56) were also found to be quantitatively highlighted, and the participants encountered the experiences of perceived barriers to cooperation in the following manner:

“*The biggest challenge is information exchange*.” P4.

Some of the respondents highlighted the importance of connecting information, reporting, and decision-making centers.

The positive correlation between awareness and implementation (*r*^2^ = 0.167, *p* = 0.001) was expressed qualitatively, specifically in the situations when participation in leadership and the support of an institution were outlined:

“*Where the leadership believes in One Health, things move faster*.” P2.

Combined, the complementary results show that concerns about implementation are less about individual attitudes than about methods of organizing coordination, accountability, and processes.

### 3.10 Expansion: qualitative findings adding depth beyond the survey

The qualitative stage expanded understanding beyond what the survey covered. Despite moderately high scores of implementations (*M* = 2.75–3.18), participants outlined the impact of the curriculum structure, the processes of accreditation, and training waypoints on the preparation of the future labor force:

“*Universities incorporating some of the training within the curriculum will be very helpful… I think we need to engage universities specifically much earlier*.” P2.

When it comes to early detection and response, the role of rural and community actors was also mentioned by the participants:

“*Farmer owners and rural communities must be engaged… they are the first to see animal diseases before anyone else*.” P1.

Besides, qualitative descriptions broadened the discourse into the direction of future system developments, such as the integration of digitalisation and predictive analytics:

“*We need a national platform that connects data… and in the future, AI can help us see risks before they spread*.” P7.

Lastly, participants had identified OH with national strategies and global agendas, and often supported more formal national coordination systems:

“*...translated into clear vocal point and some measurable… deliverables that could actually measure how well we work with this entity?*” P2.

The expansion results show that qualitative inquiry not only elucidated the quantitative tendencies but also revealed other areas of exploration, such as education systems, community involvement, and digital futures, that are core to how OH should be operationalized but are not captured in standard surveys.

### 3.11 Overall integration interpretation

This clarifying sequential design enabled the qualitative phase to expand, contextualize, and generalize the patterns identified in the quantitative survey. As part of the integration mechanism, side-by-side comparisons and joint displays (Table 10) were conducted to identify convergence, complementarity, and the expansion of results. The overlap of both phases indicates a system transition with great conceptual fidelity and deepened institutional involvement, but with continuing areas of improvement in governance, technical command, and operational streams. These combined perceptions directly influenced the innovation and growth of the suggested national OH structure.

**Table 10:** Joint display integrating quantitative and qualitative findings.

Quantitative finding	Qualitative explanation	Interpretation	Outcome of integration
81% reported no prior OH knowledge; collaboration score low (*M* = 0.51)	“Coordination between other entities depends on the nature of the work. Some diseases have their own dedicated committee…” P3	Collaboration occurs but tends to be episodic and situational.	Convergence
Correct attribution of zoonotic responsibilities 15–26%	“Each sector has the authority and the right to set its own priorities and develop its own programs… We aligned both plans… and we meet at the end of each quarter or each year… to review what has been accomplished.” P3	Uncertainty regarding institutional roles reflects.	Convergence + Explanation
Lowest awareness in AMR and environmental toxicants (*M* = 2.62–2.79)	“AMR is also overseen by a multisectoral committee that includes environment, animal health, and public health.” P3	Cross-sector issues tend to have lower visibility, especially when ownership is unclear.	Expansion
Awareness is positively associated with implementation (*R*^2^ = 0.167)	“Where the leadership believes in One Health, things move faster.” P2	Leadership engagement and capacity appear linked to practice.	Convergence
Existence of institutions handling environmental emergencies (*M* = 3.18)	“All of them are active. Maybe in past periods, the environment sector… its engagement was limited in some tracks — not across all One Health domains.” P3	Variability in stakeholder engagement levels appeared to vary across sectors and over time.	Convergence + Expansion
Poor agreement across all zoonotic diseases (κ = −0.13 to −0.10, all *p* < 0.001)	“.translated into clear vocal point and some measurable… deliverables that could actually measure how well we work with this entity?” P2	Stakeholders recognized the need for clearer shared coordination mechanisms.	Complementarity

### 3.12 Reflection of the current One Health configuration in Saudi Arabia

As perceived by the participants, OH is already implemented in Saudi Arabia through a multiple issue and sector-based arrangements, however, these efforts do not currently operate within a unified national operational framework. Interviewees described a variety of coordination strategies, including technical committees addressing antimicrobial resistance and vector-borne diseases, as well as maintaining surveillance, laboratory, and response capabilities at individual ministries and authorities.

Participants did not described an absence of OH activity, but rather the absence of a clearly defined operational structure capable of integrating these activities across sectors, risks, and governance levels. Coordination was often described as episodic and mobilized during outbreaks or priority programs, relying heavily on committees, interpersonal communication, and informal coordination pathways rather than institutionalized system-wide integration.

The findings suggest that Saudi Arabia currently demonstrates important foundational OH capacities; however, governance pathways, data-sharing systems, accountability structures, and multisectoral operational integration remain under development.

### 3.13 The proposed One Health operational development framework in Saudi Arabia

The proposed framework presents a multi-level operational model designed to strengthen governance, coordination, information sharing, and implementation of OH activities in Saudi Arabia. This framework was developed as a context-specific operational governance model informed by integrated quantitative and qualitative findings, stakeholder experiences, and national institutional structures.

The framework components have been informed by the findings of this study, mainly by the noted gaps in coordination within institutions, data fragmentation, unclear sectoral roles, limited educational integration, and the need for formalized multisectoral governance mechanisms.

Strategically, international and regional organizations offer technical expertise in line with WHO, FAO, WOAH, and UNEP’s recommendations, while national-level entities develop these priorities within the context of Saudi Vision 2030 and the Sustainable Development Goals (SDGs).

In the central of the framework is a proposed National One Health Coordination and Data Hub responsible for supporting multisectoral coordination, shared reporting pathways, integrated surveillance systems, interoperable data exchange, dashboards, and early warning functions. The framework also proposes formal communication pathways and technical coordination mechanisms between ministries, universities, laboratories, and frontline sectors.

At the operational level, institutional actors contribute through surveillance, reporting, outbreak investigation, laboratory integration, workforce collaboration, and coordinated response activities. While community stakeholders, frontline practitioners, veterinarians, inspectors, and environmental actors contribute to local detection, reporting, and implementation processes based on participation, and shared responsibility.

The framework is rely on three central OH domains of human, animal, and environmental health. That supporting the governance, financing, sustainability, regulations, digital systems, research and innovation, workforce development, and resilience. These operational components are strengthened through the 4Cs principles that include cooperation, communication, coordination, and capacity building.

The framework structures support measurable OH outputs, including shared outbreak simulations, integrated surveillance, antimicrobial stewardship programs, interoperable digital dashboards, workforce training, and multisectoral pilot projects.

## 4 Discussion

This study examined OH awareness, implementation, and the challenges associated with them. The study included both early-career health-related participants and senior OH stakeholders. The combined conclusions state that, despite conceptual alignment with OH, the system is still consolidating governance-related arrangements, technical infrastructure, and education pathways required to enable routine multisectoral coordination and implementation.

### 4.1 One Health awareness

In line with the empirical findings of other researchers, OH remains a relatively emerging concept outside specialist communities; the general awareness of the construct within this sample was limited despite its high perceived value (18). The vast majority of respondents reported no prior knowledge of OH but strongly supported its usefulness for disease control, health and wellbeing, research, and international cooperation (4, 28). This trend is similar to the description of OH as a growing yet more legitimized trend worldwide, combining human, animal, and environmental health to address zoonoses, AMR, food safety, and environmental risks (6).

The understanding was much stronger among males, older age, those with higher levels of postgraduate training, and academic professionals, suggesting that the general level of OH understanding is clustered among more professional or experienced populations. Along these lines, other studies have also reported identical patterns that OH awareness rises with seniority and is generally higher among veterinary and health students than among other students (24, 29, 30). These rather low levels of awareness among undergraduate and nursing students may indicate that subsequent workforce generations will not be well exposed to OH concepts in formative training, reflecting the needs assessment of the lack of cross-training of medical, veterinary, and environmental students in OH and public health (31). Although statistically significant, the regression models explained only a limited proportion of variance in awareness scores, suggesting that additional institutional, educational, and contextual factors may influence OH awareness beyond demographic characteristics alone.

The modest positive association between awareness and self-reported implementation provides a basis for interpreting knowledge as an enabling factor. Researchers have found that, as in other educational and workforce contexts, those with more positive knowledge of OH are more likely to engage in collaborative practices, appreciate cross-sector problems, and identify appropriate structures (32). This underscores the need to incorporate OH material into the curriculum, accreditation standards, and pre-professional training, as reflected by qualitative informants and reinforced by newer educational paradigms that recommend incorporating OH into health professional curricula (33).

### 4.2 Implementation and collaboration

Whereas the survey scores on the OH implementation scale and collaboration were relatively central, qualitative descriptions added depth with respondents characterized collaboration as relatively strong in selected areas but mainly episodic. Personal networks often mediated coordination and could help to solve problems quickly, as they stated. Similar patterns have been observed in the Eastern Mediterranean Region, where OH implementation is underway but unevenly distributed, and where multisector collaboration has mostly been stimulated by particular events or leadership projects rather than institutionalized mechanisms (34, 35).

In institutions where leaders are actively supportive of OH, implementation proceeds more quickly, as reported. This aligns with previous OH implementation studies highlighting the importance of political commitment, institutional leadership, and formal governance structures in sustaining multisectoral collaboration (4, 5, 36).

### 4.3 Governance and role clarity

The survey results indicated low levels of agreement and negative kappa values in attributing responsibility for zoonotic diseases, suggesting a lack of common understanding of the role among ministries. Qualitative data made it clear that this is contingent on changing coordination arrangements, rather than a total lack of structure, as sectors maintain their mandates and regulatory functions and coordinate through joint planning and review. This is consistent with the national evaluations of the country, which have laws and standard operating procedures in place for zoonotic disease priorities and have implemented OH-compatible policies (37).

Also, world issues and complex OH challenges may fall victim to scattered ownership and polarized responsibility if leadership and coordination mechanisms are not properly established (38). The Quadripartite OH Joint Plan of Action (OH-JPA) specifically recommends the establishment of enhanced national governance platforms to define roles, harmonize mandates, and operationalize shared responsibility across the human, animal, plant, and environmental sectors, which are closely reflected in the proposed framework of action in this study targets (5).

### 4.4 Information systems and technical capacity

Some of the most prominent constraints were information sharing and data interoperability. Respondents explained the use of interpersonal channels to receive data and demand integrated digital platforms, dashboards, and further use of AI to detect risks in the future. Such experiences are reflected in the list of priorities developed by WHO and partners, which focus on interoperable surveillance systems and integrated risk assessment to operationalize OH and accomplish OH-JPA action tracks (4, 5).

The monitoring of surveillance, laboratory capacity and preparedness to priority zoonotic diseases and AMR in Saudi Arabia has already received significant investment. The current results indicate that the next step will also require both technical facilities and governance agreements and processes that enable the daily flow of information across industries and levels (39, 40). This is congruent with the new recommendations to interconnect OH capacities with other health systems strengthening and the digital transformation agenda (5, 10).

### 4.5 Underused actors and domains

One of the contributions of this study is to shed light on aspects rarely examined in discussions of OH implementation. Academic institutions are theoretically supportive of OH but less responsive to emerging multidisciplinary agendas. This This observation aligns with remarks that universities are drivers of OH change through curriculum reform, improved interprofessional education, and practitioner training (41, 42).

Also, participants stressed the importance of rural communities and farmers as the early signalers of animal and environmental changes. These actors have resonated with other analyses that call for more participatory, contextually sensitive, participatory decision-making processes by OH in Arab and EMR (43).

The increasing involvement of environmental sectors reflects broader shifts within OH agendas toward climate, biodiversity, and sustainability integration. The study broadens the discussion of classical agendas on zoonotic and antimicrobial resistance. It improves the integration of the OH into other efforts to combat climate change, biodiversity, and sustainable development by surfacing unexplored actors and domains (44, 45).

### 4.6 Implications of the proposed National One Health Framework

The joint findings help to promote a realistic vision of the OH system in Saudi Arabia. The results indicate a system in transition rather than a lack of OH activity, as legislation, surveillance capabilities, and cross-sectoral efforts exist. Nonetheless, the clarity of governance, data, and educational and community connections requires stronger coordination, integration, and formalization mechanisms (5, 37).

The operational model in this paper, which incorporates a National One Health Coordination and Data Hub, 4Cs (Cooperation, Communication, Coordination, Capacity building) and balanced representation of human, animal, and environmental sectors, has a lot in common with the OH JPA and the EMR guidance, which proposed high-level coordination, integrated information systems and multisectoral workforce development (5, 34).

The framework serves as an organizing prism for national priorities in health security, food systems, environmental protection, and digital transformation, explicitly connecting OH to Vision 2030 and the SDGs (46). Unlike conceptual OH models, the proposed framework emphasizes operational coordination pathways, accountability mechanisms, interoperable information systems, and implementation structures tailored to the Saudi context.

### 4.7 Strengths and limitations

To the best of our knowledge, it is one of the earliest mixed-methods assessments of OH awareness and system preparedness in Saudi Arabia, integrating a large survey of current and future health workforce members and qualitative data from senior stakeholders. The explanatory sequential design enabled a framework for national integration to propose the mentioned gaps and challenges.

Nonetheless, the sample was skewed toward female and undergraduate students at academic institutions. Therefore, the findings should be interpreted primarily as reflective of university-affiliated and early-career populations rather than the entire national OH workforce. In addition, awareness and implementation measures relied on self-reported responses and may therefore be influenced by social desirability or recall bias. Finally, the cross-sectional design captured perceptions at a single point in time, whereas OH systems and institutional structures in Saudi Arabia continue to evolve; therefore, longitudinal studies are recommended to assess changes over time.

## 5 Conclusion

This study demonstrates that OH implementation in Saudi Arabia is evolving, although formal awareness and operational integration remain uneven across sectors and workforce groups. The formal OH concept is still poorly known to students and some professional groups, but it is perceived as having high value in implementation activities. The collaboration exists, with suggested enhancements in governance, data sharing as a challenge in information systems, and involvement with underrepresented partners.

These recommendations underline the creation of a national operational framework of OH that creates a mandated coordination and data hub; has proven governance, accountability, and financing; invests in interoperable digital surveillance and analytics; encompasses OH competencies in education, training, and accreditation; and coordinates systematically the engagement of communities and underrepresented sectors. It can be expected that such a structure, which is consistent with global OH guidance and Vision 2030, may support the transition of OH in Saudi Arabia from conceptual endorsement toward more coordinated operational implementation and preparedness.

Further studies are advised to track the deployment of the suggested frameworks, evaluate their effects on specific outputs (e.g., outbreak response, antimicrobial resistance patterns, curriculum change), and, in more detail, focus on the functions of plant, environmental, and community actors.

## Data Availability

The raw data supporting the conclusions of this article will be made available by the authors, without undue reservation.
